# Family-Centered Care in Neonatal Intensive Care Unit: A Concept Analysis

**Published:** 2014-10

**Authors:** Tahereh Ramezani, Zahra Hadian Shirazi, Raheleh Sabet Sarvestani, Marzieh Moattari

**Affiliations:** 1 Student Research Committee, Shiraz University of Medical Sciences, Shiraz, Iran;; 2 Department of Nursing, Fasa University of Medical Sciences, Fasa, Iran;; 3 Department of Nursing, School of Nursing and Midwifery, Shiraz University of Medical Sciences, Shiraz, Iran

**Keywords:** Family Centered Nursing, Neonatal Intensive Care Units, Rodgers’s Concept Analysis

## Abstract

**Background:** The concept of family- centered care in neonatal intensive care unit has changed drastically in protracted years and has been used in various contexts differently. Since we require clarity in our understanding, we aimed to analyze this concept.

**Methods:** This study was done on the basis of developmental approach of Rodgers’s concept analysis. We reviewed the existing literature in Science direct, PubMed, Google Scholar, Scopus, and Iran Medex databases from 1980 to 2012. The keywords were family-centered care, family-oriented care, and neonatal intensive care unit. After all, 59 out of 244 English and Persian articles and books (more than 20%) were selected.

**Results:** The attributes of family-centered care in neonatal intensive care unit were recognized as care taking of family (assessment of family and its needs, providing family needs), equal family participation (participation in care planning, decision making, and providing care from routine to special ones), collaboration (inter-professional collaboration with family, family involvement in regulating and implementing care plans), regarding family’s respect and dignity (importance of families’ differences, recognizing families’ tendencies), and knowledge transformation (information sharing between healthcare workers and family, complete information sharing according to family learning style). Besides, the recognized antecedents were professional and management-organizational factors. Finally, the consequences included benefits related to neonate, family, and organization.

**Conclusion:** The findings revealed that family centered-care was a comprehensive and holistic caring approach in neonatal intensive care. Therefore, it is highly recommended to change the current care approach and philosophy and provide facilities for conducting family-centered care in neonatal intensive care unit.

## Introduction

More than 400000 American families undergo to have premature neonates annually. The statistics in Iran showed that 6.5% of neonates were low birth weight among which, 75% were premature and needed to be hospitalized in Neonatal Intensive Care Unit (NICU).^1^This experience threatens not only neonates’ health, but also family members’ health and brings a vast array of problems. Hospitalization of premature neonates in NICUs for a long period of time causes the family to be isolated from their neonates and changes their hope into anxiety. Then, parents may state that they do not have any parental feelings and cannot communicate with their neonates. This causes a delay in parental-neonatal attachment (especially for mothers) and upsets mothers’ attachment process toward their babies. Moreover, some factors, such as complexity of units facilities and technology, hospital policies and missions, neonates’ behavior and appearance, and inability to support the baby, induce feelings of guilt, fear, anger,  loss, inability, and hesitation in family members.^2^^,^^3^ As a result, significant advances in taking care of premature neonates have been achieved in the recent decades. For the first time, Bowlby and Robertson (1960) stated the negative consequences of neonate hospitalization in NICU and its adverse effects on premature babies and their families. Besides, they considered family as a unique factor which played an important role in taking care of the neonate.^4^ The impact of intimacy and attachment theories on neonates’ growth led to development of new policies by health and medical departments which changed the medical team members’ attitude towards involving families in neonates’ care plans.^5^ Up to now, great attempts have been made to use care approaches and models for supplying neonates and families’ needs. In this regards, Family-Centered Care (FCC), as a paradigm, care model, philosophy, or theory, has been considered by health and medical departments practically.^4^Heidelise et al. (1970) planned developmental care for hospitalized neonates individually. They provided these cares through observing neonates’ behaviors, as a practical guide. This led to progress in FCC in which neonates were regarded as individual beings.^6^ In fact, neonate-centered cares and task-directed cares have changed into comprehensive ones on the basis of individual variations and communication principles.^7^ FCC, as a medical concept, is a golden standard in NICU. This approach, as a team-oriented and multi-disciplinary one, involves families in breastfeeding, kangaroo care, cares planning, and limitless presence beside their neonates. In addition, it enables the family members to take care of their neonates with less expenses and optimal quality.^8^ Researchers have considered FCC as a part of supplementary protection. The advantages of FCC for health organizations include saving expenses in the long run due to shortening the duration of neonates hospitalization in NICU, decreasing the prevalence of some contradictions such as different kinds of infections, and reducing neonates’ re-hospitalization.^9^

However, FCC in NICU has different definitions and descriptions in various resources with diverse goals. In fact, this concept has been changed and developed drastically since 1993 and other concepts have been added to this concept gradually.^2^^,^^10^^,^^11^ Even considerable controversies were created among professionals when it came into be used practically, and studies have shown different understandings of FCC according to where it has been applied.^4^ Although different studies have proved the efficacy of this concept in solving problems in NICU, its various definitions have made a great deal of challenges in using this approach in practice. Yet, recognizing all the aspects of a concept closely facilitates its comprehensive recognition. In contrast, if this does not occur especially in NICU, healthcare team will get confused in practice. Therefore, it is mandatory to analyze this concept in NICU. Although Malusky et al. (2005) performed a concept analysis on FCC, they did not completely explain about its method.^12^ Furthermore, concepts have a dynamic nature and are not constant over time; thus, we aimed to analyze this concept and it is possible to analyze it in future, as well. There are diverse ways to analyze concepts related to practice in nursing. Rodgers’s developmental concept analysis is an inductive method. He believed that concepts are developed over time and are affected by fields in which they are applied. Hence, concepts are developed continually and are refined dynamically.^13^ Rodgers (2012) believes that concepts should be purposeful and practically used and also have to participate in solving problems and provide necessary characteristics of the issue.^13^The present study aims to analyze FCC in NICU using Rodgers’s developmental approach. The characteristics, antecedents, and consequences of the concept are going to be determined, as well.

## Materials and Methods

Rodgers’s developmental concept analysis**, **as an inductive method in analysis, was used in this study. He noted some steps for his concept analysis (table 1), but emphasized that this process was not linear and consisted of some steps with similar characteristics. The first step in concept analysis is to choose the interested concept. After determining the concept, the number of the necessary articles for data collection should be determined. In the current study, English references with full text formats about FCC in NICU in nursing, medical, and psychology fields were searched from 1980 to 2012. Since the enlighten period of this concept and its impact on neonates’ health was supposed from 1980, this year was chosen to begin our search. The databases used for searching included Science direct, PubMed, Google scholar, and Scopus. Besides, FCC, family-oriented care, and NICU were used as keywords in either the titles or the abstracts. After all, 244 nursing and 2 medical articles (that were not repeated in these databases) were found. Then, more than 20 percent of these articles, including 50 nursing and 2 medical ones (that were more related to our concept), were selected. Also, FCC and NICU keywords (in Persian) were searched in an Iranian database called Iran Medex and five articles, as sample cases, were obtained. Manual search was also performed in the library of the School of Nursing and Midwifery in Shiraz. Accordingly, Wong’s book, as a reference, and a thesis which was related to FCC in NICU were selected. Overall, the sample consisted of 59 English and Persian articles in nursing and medical fields. The selected articles were read carefully to collect the study data. Rodgers and Knafl (2000) stated that every researcher uses his/her individual method for collecting, organizing, and monitoring data, but he/she must determine clearly the study goals and applied analysis techniques.^14^ In the present study, qualitative content analysis was used to analyze the data. At first, open coding was used to determine the characteristic, antecedents, consequences, substitution words, related concepts, and FCC definitions in NICU. Then, open codes, written on pieces of paper, were read for several times by the researchers to determine the categories. This was done as precisely as possible. After determining the categories, the primary codes were put in the related cluster by continuous comparison, and abstraction was done. Since Rodgers supported writing a real model case about the concept, a real model case of FCC in NICU was written, too.

**Table 1:** Rodgers’ (1989) method for concept analysis

**Steps**	**Definition**
1	Identify the concept of interest and associated expressions (including surrogate terms).
2	Identify and select an appropriate realm (setting and sample) for data collection.
3	Collect data relevant to identify.
4	Analyze data regarding the above characteristics of the concept.
5	Identify an exemplar model case of the concept if appropriate.
6	Identify implications, hypotheses, and implications for further development of the concept.

## Results

Our results showed the attributes, antecedents, and consequences of FCC and the related concepts, substitution words, and selected examples were identified simultaneously. Table 2 shows the summary of the reviewed articles about FCC concept in NICU.^3^^,^^5^^,^^15^^-^^17^

**Table 2:** The summary of the reviewed articles about family centered care concept in neonatal intensive care unit

**Author **	**Attributes**	**Antecedents**	**Consequences**
Trajkovski et al., 2012^15^	Family centered care in NICU is a method to provide healthcare for neonates and their families. These cares should be planned for all family members.	Changing nurses’ role from a skillful one into a guide, system design, use of policies, staff’s education, multi-disciplinary approach application, limitless contact, providing care for neonates and families, nurses’ need assessment, developing efficient and trust-based communication, family participation in daily cares, data exchange, guiding the family, guiding the staff, supporting nurses, nurse commitment	Promoting neonates’ health criteria, power improvement, increasing parents’ ability in taking care of neonates, staff’s professional improvement, reinforcing neonate-parents contact, successful performance of Kangaroo care plan
White, 2010^16^		Providing a single room for family, making policies, enough space, providing more expert staff and financial sources.	Improving parents’ interaction with neonate, infection control, discharging the neonate sooner than usual, affecting neonates’ brain development, increasing parents and staff satisfaction, parents’ competence in care giving to neonate, feeling of being at one’s own home, reducing healthcare expenses, improving safety.
Hedbeg and Egvall, 2009^5]^		Parents’ feeling of responsibility, providing policies, considering a single room for parents and their baby, improving individual care attitude in parents, gradual education of parents regarding care, changing nurses’ role from care providers into trainers, providing weekly care plans, parents’ right to choose.	Increasing neonate-parents attachment, feeling of intimacy towards the neonate.
Griffin 2006^17^	Family-centered care in NICU is an approach to planning, implementing, and evaluating healthcare which is formed on the basis of participation of different health and family professions. Its four basic principles are dignity and respect, information exchange, family participation in care, and family cooperation.	Existence of family centered care philosophy statement and outlook, single room, improving nurses’ communication skills, presence of parents in all conditions, encouraging the parents, educating the parents on neonatal care skills.	Decreasing parents’ stress, increasing mothers’ self esteem, promoting neonate’s health, improvement of parental process, reinforcing parents’ mood, inducing a sense of control in parents.
Van Riper 2001^3^	Family-centered care in NICU is an interaction among family members, nurses, doctors, and other health providers.	Cooperation among family members and healthcare providers, reporting neonate’s condition to parents, exchanging information about cares with parents, replying all parents’ questions, positive relationship between parents and healthcare team.	Parents’ mental health promotion, feeling of belonging to NICU, parents’ trust in healthcare providers.

*Attributes*

One of the attributes of FCC concept in the present studywas “family’s care taking”. This attribute composed of assessment of family and evaluation and provision of its needs. The second attribute of FCC conceptwas “equal family participation”**. **This attribute involved family participation in care planning, decision making, and providing routine and special cares. The third attribute of the FCC concept was “collaboration”. This attribute included inter-professional collaboration with the family and its involvement in regulating and implementing of care plans. The forth attribute of the FCC concept was “maintaining family’s respect and dignity”.  This attribute had two specifications, including the importance of families’ differences and recognizing their tendencies. The last attribute of the FCC concept was “knowledge transformation”. This attribute included information sharing between healthcare workers and families and complete information sharing according to families’ learning styles.

*Antecedents*

The antecedents of FCC were classified as professional and management-organizational factors. The subcategories related to professional factors consisted of families’ individual characteristics, professionals’ skills, and care providers’ attitudes towards FCC. On the other hand, the subcategories related to management-organizational factors composed of integration in management, providing the philosophy of FCC, and supplying educational sources.

*Consequences*

FCC consequences in the present study consisted of those related to neonates, families, and organizations. The consequences related to neonates were divided into short- and long-term outcomes. The short-term consequences were decreasing neonates’ hospitalization and re-hospitalization in NICU, promoting their connection to their parents, increasing deep sleep episodes and safety, supplying all their needs, relieving pain, controlling restlessness, and reducing the use of analgesics. On the other hand, the long-term outcomes consisted of improving neonates’ behavioral and nervous development, diminishing physical and emotional abuse, and positive impacts on neonates’ learning.Theconsequences related to families included increase of emotional well-being and adjustment to the conditions, improvement in self-esteem and independence, sense of control, and supplying needs. Besides, the perceptual and functional outcomes for families included enhancing decision-making and responsibility, logical understanding of neonates’ conditions, obtaining sufficient information, gaining qualification in care giving, ability to provide permanent care at home, responding to neonates’ needs, and being satisfied by medical cares.Finally, theconsequences related to organizations consisted of economical and medical team-related outcomes. The first one led to reduction of the expenses paid for therapy, while the latter resulted in promotion of social skills, inter-professional cooperation, qualification and capabilities, professional satisfaction, and increase of knowledge about care. Gaining a criterion for permanent evaluation of professional qualification was another organizational consequence in this study.

*Definition*

Recognition of concept characteristics is the first step of analysis which leads to actual definition of the concept. Some FCC definitions in NICU have been illustrated in table 1. In the present study, FCC is defined as an inter-disciplinary, comprehensive, and holistic care of neonates and families with maintaining their respect and dignity. Family, as a constant member in neonate’s life and one of the main participants in healthcare, collaborates mutually with healthcare workers. Complete information exchange without any bias leads to promotion of quality of cares provided for neonates and their families.

*Related Concepts*

Application of the related concepts in analyzing the concept is based on this belief that every concept makes a network of some other ones which describe the importance of the intended concept. In fact, related concepts are a part of the connections of the main one, but do not have all attributes of the intended concept.^14^ The most common keywords used in analyzing the reviewed studies which had very close relation with FCC were developmental care, family centered developmental care, individualized family centered developmental care, individualized nursing intervention, and family provider relationship. In addition, surrogate words were those which were similarly defined to the intended concept in the reviewed studies. This means that the concepts’ meanings were stated by other words.^13^^,^^14^ The results of process analysis showed that FCC concept in NICU could be replaced by some phrases, such as family service care, family support care, and family-oriented care.  An example of the attributes of the concept has been presented in table 2. According to Rodgers, concept analysis could include a real model case. In this study, the researchers found such a case which has been presented below:

*Mason and Grady were born in the 24^th^ week of gestation with 5.1 pound weight in 2008. They were transmitted to children’s hospital just after being born. I got discharged from the hospital in less than 24 hours and referred to children’s hospital. My husband gave me some information about our babies and my expectations. I was aware of hospital atmosphere and NICU to some extent due to my profession; therefore, I was not scared of any unknown thing. When we came in, nurses, doctors, and other personnel were present to answer our questions. When one of my sons was about two or three days old, a nurse gave us a good book. We found the sense of control over the situation by gaining some more information and reading that book. On the first days, the best gift which the nurses gave us was to let us touch our babies, change their diapers, and any other thing leading to more attachment with them. This made us to feel parenting and participating in cares. I took care of my babies with nurses and was informed about their time limitation and all the procedures they did. On the tenth day, Mason was caught by cerebral hemorrhage and his attempt to survive ended just three days later. The most difficult decision in our life was to let staffs disconnect the machine. They respected our decision and supported us. Our sorrow was so dramatic, but we had to go on taking care of Grady. It is very sorrowful to come into a unit in which one of your babies is not there anymore. The nurses not only took care of our baby, but also supported us emotionally. Judy, a primary nurse, Marian, a clinical nurse, and I understood each other. The presence of the nurses working in fixed times led to persistent qualified cares. Judy and I planned for the next day every day. Whenever my husband and I arrived in the ward, Marian or one physician accompanied us to Grady’s bed. They let us know how he was. Grady’s condition got better gradually. I remember vividly when he could be milked by bottle. Judy, a nutritionist, and I tried to schedule Grady’s feeding time according to my own work timetable. We got ready to take Grady to our own home on the last day of January, but his condition got worse abruptly. His breath was blocked and his skin color got blue during feeding. The nurses and doctors arrived in the room and resuscitated him. I looked at them. A nurse stood next to me, took my hands, and described something to me. I did not remember what she said, but I found that I was not alone. Grady was taken home in March 2009 with oxygen, monitoring machines, and a lot of medications. The therapies were continued and the physicians evaluated his condition carefully. Grady is 3 years old now and ok. All staff of NICU are the main agents of our family survival. The staff let us participate in taking care of our elder child. They took care of our family while doing the same for our admitted baby *.^18^

## Discussion

In this study, Rodgers’s developmental approach was used to analyze FCC in NICU. In this part, the characteristics, antecedents, and consequences of FCC are discussed and compared to the literature. 

Concept attributes facilitate recognizing the situations which can be categorized on the basis of the concept.^15^One of the concept attributes of FCC in the present studywas “care taking of family”. This attribute consisted of assessment of families and their needs and providing their requirements. FCC has been recognized, in different studies, as the best qualified way to determine families’ mental and physical problems and their needs. Nurses and other members of the healthcare team try to bring about qualified care plans for all family members (not only neonate), as the receivers of the cares.^15^ Frank and Callery (2004) believed that finding their needs enabled family members to adjust themselves to this situation and the medical unit. Besides, they could gain control over their behavioral and emotional reactions. These consequently led to improvement of psycho-social function, decrease of stress, enhancement of satisfaction with the received services, and reinforcement of the family members’ capabilities.^19^

The secondconcept attribute of FCCwas “equal family participation”**. **This attribute included family participation in care planning, decision making, and providing cares from routine to special ones. McGrath (2011) stated that FCC was a kind of inter-professional care in which the families participated in the routine cares based on the care philosophy focusing on the specific needs of the family members. They even participated in medical rounds, emergencies, doing simple to more special procedures, and nursing reports. As parents keep in touch with the medical staff and take part in planning, regulating, and implementing the care programs, they will be able to play a crucial role in making significant decisions for their own babies.^6^^,^^20^ The results of studies also showed that families considered their participation in care plans as an efficient factor in achieving the capabilities to take care of their babies, make qualified decisions, and feel more responsible toward them.^21^

The third concept attribute of FCCwas “collaboration”. This attribute included inter-professional collaboration with family and their involvement in regulation and implementation of care plans. According to Hendricks, FCC focused on multi-disciplinary and team work approach.^22^Creating a collaborative environment through involving families in policymaking and planning for activities and educational programs together with health organizations’ managers and medical team can lead to improvement of the care programs that facilitate FCC advancement.^17^

The forth concept attribute of FCC was “maintaining family respect and dignity”.This attribute had two subcategories including the importance of families’ differences and recognizing their tendencies. FCC regards family members as the most significant source of supplying neonates’ growth and socio-emotional needs. It also considers families’ culture and beliefs to develop care principles.^17^^,^^20^ Respect, variation in understanding, supporting families’ preferences, considering freedom, honesty, and neutralization are the other characteristics of FCC.^21^ McGrath (2011) believed that FCC philosophy did not include carrying out the predetermined policies and planning cares for patients and families without considering their tendencies and variations of their individual capabilities and capacities.^6^

The last concept attribute of FCCwas “knowledge transformation”**.** This attribute included information sharing between healthcare workers and family and complete information sharing according to family’s learning style. Although parents should be trained specifically and given enough data on the basis of their learning style, they themselves are a rich source of information and experiences in FCC; therefore, data exchange is bilateral.^23^ Review of the literature showed that nurses in FCC applied their knowledge, information, and skills to enable and inform families regarding neonatal care through role playing, educational roles, consulting, and care giving methods.^15^ This data exchange was done in accordance with mental abilities and functional capabilities of families through different styles (e.g. visual and auditory) and methods (e.g. writing and electronic instruments). The level of family perception must be taken into account, as well.^5^ Overall, effective data exchange between medical team and family members for empowering the family, making sense of control, decision making, taking responsibility, and achieving a realistic view towards their neonate’s appearance and health condition played an important role in FCC.^19^^,^^20^

Antecedents are defined as the events and phenomena which were related to the concept in the past. At this stage, researchers’ question is “what happened before the concept”. Rodgers (2000) suggested that review of the literature had to identify the antecedents and consequences of a concept, because both of them help clarify the concept.^13^^,^^14^ In the present study, the antecedents of FCC were professional and management-organizational factors.The results of studies showed that the first step in FCC implementation was changing attitude, behavior, and perception of professional individuals in order to get family involved in care, clear identification of the roles, and individual responsibilities. Lack of appropriate background and negative attitude leads to ineffective communication, loss of respect and acceptance, limited and inflexible instructions, getting family away from the neonate, and performing traditional and neonate-centered care.^24^^,^^25^ Nonetheless, providing FCC results in promotion of the services quality. Nurses’ responsibility is to interact with family members and enable them as far as possible to make positive changes on the basis of their individual capabilities. They also have to create an atmosphere in which family members can use their own abilities and personal characteristics. Hence, they will be able to take care of their neonates and gain the necessary capabilities.^15^ FCC emphasizes that family can soon achieve the ability to provide care by nurses’ aid. It has been supposed that they are able to adjust themselves with neonates’ conditions and the unit in which they are admitted.^5^Yet, nurses’ skills and qualifications affect the efficiency of FCC. Thus, they must raise their knowledge through qualified education and evaluation of their performance. This will result in enabling and training families to get involved in cooperative cares. In fact, FCC philosophy considers the impact of family on neonate’s health, and health organizations should support families to have a positive effect on neonates’ health.^26^ Therefore, providing the philosophy, landscape, and practical standards used in FCC must be done in health organizations. The policies, performances, and care plans should also be adopted on the basis of the philosophy.^6^Ballweg (2001) suggested that family-oriented care committee was established in health organizations. This can be a footstep for developing family participation, team working, and identifying the expectations in accordance with standards and family culture. The responsibilities of this committee consist of assessing physical environment (sound, light, appearance, and family private territory), evaluating the philosophy (emphasizing participatory care method, inter-professional communication, family guidance and support, and individual care), monitoring care providers (observation, support, and education), and cooperation among cares (team working, time tabling, preferences regulation, and transformational support).^7^ Moreover, flexible management is necessary to solve financial problems and lack of personnel and facilities, regard nurses’ independence and respect, and rely on them.^25^ Therefore, changing organizational policies and professional education system whether in clinical or training fields is very crucial to provide qualified FCC.

Another part of the study was related to consequences. Consequences are defined as the outcomes of applying the concept in a practical situation. It is a phenomenon which is expected to happen after occurrence of a concept.^13^^,^^14^ FCC consequences in the present study consisted of consequences related to neonates, families, and organizations. Nichols (2012) believed that the crucial factors which could lead to prosperity of FCC were neonate-parent attachment, involvement of parents in care, and application of a supportive, friendly, and calm environment. In fact, FCC consequences enable parents to take care of their babies and begin the process of neonate-parents connection.^10^Fegran (2008) stated that implementing FCC led to earlier discharge from hospital, decreased re-hospitalization, and improved breastfeeding and parent-neonate attachment.^27^ Besides, White (2010) indicated that family oriented care resulted in parents’ satisfaction and gaining qualification to take care of neonates. This kind of care causes parents to feel that they are at their own home.^16^

Our study described FCC concept based on its antecedents, attributes, and consequences. This new definition considers FCC as an interdisciplinary and comprehensive care for neonates and families by maintaining their dignity and respect and mentions family as an constant member in neonates’ life. In this kind of care, family members and healthcare teams have equal participation and collaboration and information will be exchanged completely and unbiased, eventually increasing the quality of care. 

The current study had some limitations. Since only 20% of all the articles were reviewed to analyze the concept, it might have been studied incompletely. In addition, use of English references, as the only Latin articles, might have formed an incomplete image of this concept. Thus, this concept is recommended to be analyzed again using hybrid method according to the impact of contextual specifications of the concept application and its practical use. 

## Conclusion

In the recent years, health organizations found the advantages of cooperation between medical team members and family for a qualified care. This can be achieved by limitless presence of family at neonates’ bedside to make positive changes in them. Decision making and the responsibility for taking care of neonates and supplying treatment at home can be gained by using the family members’ capabilities. Parents can recognize their own babies’ behaviors well and take care of them with their endless affection and attention. Finally, their needs provide the basis to design care plans, so that qualified cares can be provided in accordance with all preferences and priorities. FCC is a common participation of the professional team and the family to promote the quality of the provided cares. Therefore, monitoring all its dimensions is necessary to develop its use immensely as a standardized care method. Overall, it could be concluded that FCC is a golden standard in health and treatment system which has not been applied perfectly in NICU in spite of its useful results. Therefore, this approach should be considered to change the attitude and performance of all healthcare professionals as well as in university students’ education plans. 
